# Improved cytodiagnostics and quality of patient care through double reading of selected cases by an expert cytopathologist

**DOI:** 10.1007/s00428-015-1738-3

**Published:** 2015-03-17

**Authors:** Chantal C. H. J. Kuijpers, Mike Visser, Daisy M. D. S. Sie-Go, Henk de Leeuw, Mathilda J. de Rooij, Paul J. van Diest, Mehdi Jiwa

**Affiliations:** 1 Symbiant Pathology Expert Centre, Alkmaar, The Netherlands; 2 Department of Pathology, University Medical Centre Utrecht, Utrecht, The Netherlands; 3 PALGA, Houten, The Netherlands; 4 Department of Pathology, Alkmaar Medical Centre, Symbiant Pathology Expert Centre, PO Box 501, 1815 JD Alkmaar, The Netherlands

**Keywords:** Cytopathology, Diagnostic accuracy, Patient safety, Quality assurance, Subspecialization

## Abstract

Double reading may be a valuable tool for improving the quality of patient care by restoring diagnostic errors before final sign-out, but standard double reading would significantly increase costs of pathology. The aim of this study was to assess the added value of routine double reading of defined categories of clinical cytology specimens by specialized cytopathologists. Specialized cytopathologists routinely re-diagnosed blinded defined categories of clinical cytology specimens that had been signed out by routine pathologists from January 2012 up to December 2013. Major and minor discordance rates between initial and expert diagnoses were determined, and both diagnoses were validated by comparison with same-site histological follow-up. Initial and expert diagnoses were concordant in 131/218 specimens (60.1 %). Major and minor discordances were present in 28 (12.8 %) and 59 (27.1 %) specimens, respectively. Pleural fluid, thyroid and urine specimens showed the highest major discordance rates (19.4, 19.2 and 16.7 %, respectively). Histological follow-up (where possible) supported the expert diagnosis in 95.5 % of specimens. Our implemented double reading strategy of defined categories of cytology specimens showed major discordance in 12.8 % of specimens. The expert diagnosis was supported in 95.5 % of discordant cases where histological follow-up was available. This indicates that this double reading strategy is worthwhile and contributes to better cytodiagnostics and quality of patient care, especially for suspicious pleural fluid, thyroid and urine specimens. Our results emphasize that cytopathology is a subspecialization of pathology and requires specialized cytopathologists.

## Introduction

There is a growing awareness that pathology diagnosis is not infallible and that diagnostic errors may lead to under- or overtreatment and thereby compromise patient safety. Double reading is a potentially valuable tool for reducing diagnostic errors and thereby improving the quality of patient care. It may reveal inaccurate diagnoses that otherwise might have led to improper or unnecessary patient management or treatment. In response to the Institute of Medicine report ‘To err is human; building a safer health system’ from 1999 [1], the American Society for Clinical Pathology (ASCP) recognized second opinion as a key aspect in the assurance of patient safety for histological and cytological diagnoses [2]. They recommended to consider second opinion in several situations, including highly critical or significant cases, problem-prone cases and cases suggested for review by clinicians [2].

Many studies focused on second opinion in diagnostic surgical pathology and reported major diagnostic disagreement rates of 2 to 28 %, mainly depending on the organ system studied [3–24]. A smaller number of studies focused on second opinion in cytopathology, of which the majority reported disagreement rates of specific organs or organ systems, predominantly the thyroid [25–31]. Few studies, however, assessed the impact of double reading on patient care for the whole subset of cytological specimens. These studies reported major disagreement rates ranging from 7.4 to 9.3 % [32–34], and second opinion diagnoses were better supported by histological follow-up than the initial diagnoses [32, 33].

Therefore, we implemented intradepartmental double reading by expert cytopathologists on January 1, 2012. Since routine double reading of all specimens would significantly increase costs, we predefined selected categories of cytology cases where the yield of double reading was expected to be highest. In this study, we assessed the added value of this expert double reading strategy. To this end, we retrospectively determined the rates of concordance and major and minor discordance between initial and second opinion diagnoses of all cytology cases reviewed by the expert cytopathologists. Furthermore, we validated both diagnoses by comparison with same-site histological follow-up.

## Materials and methods

### Routine intradepartmental second review

**Fig. 1:**
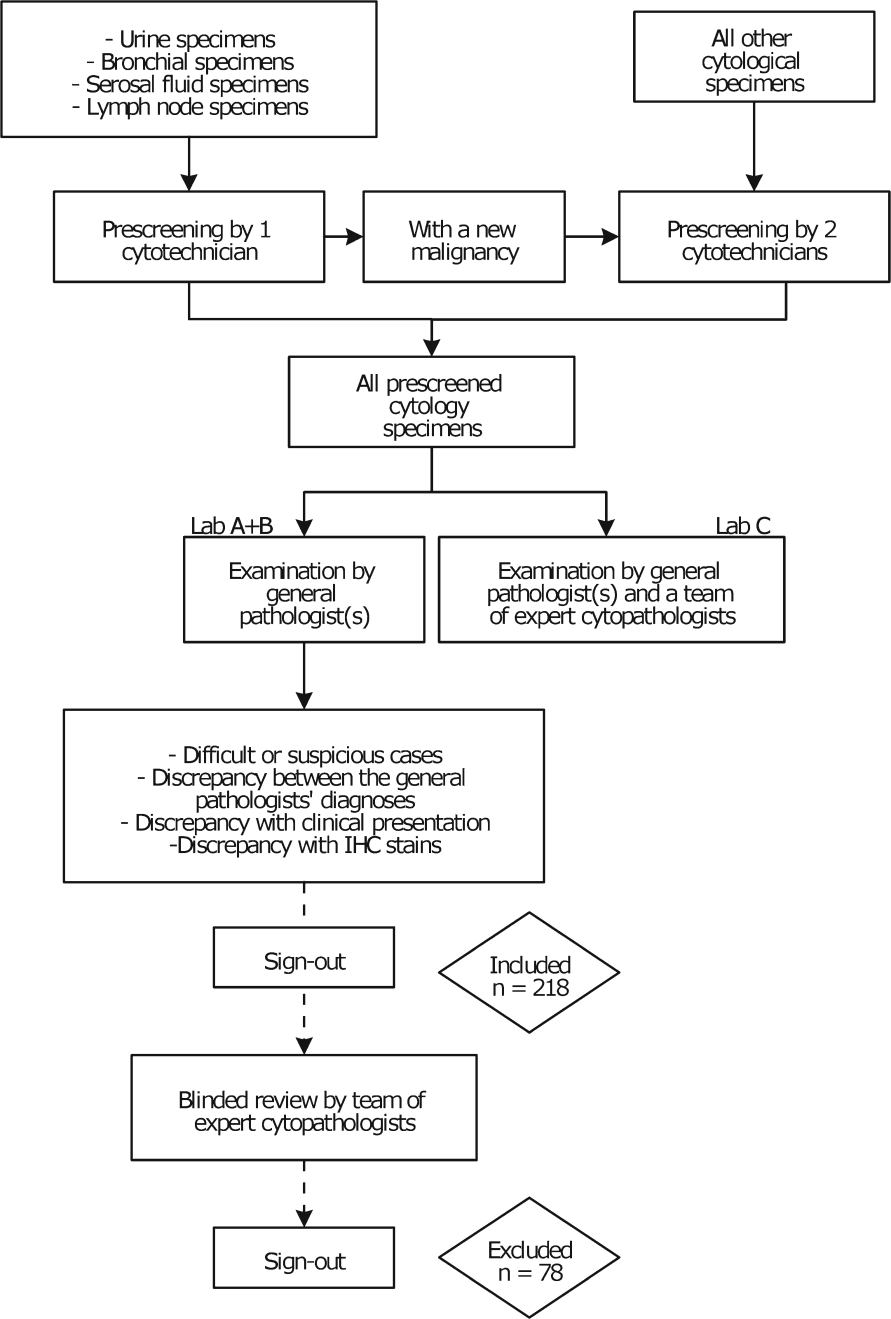
The routine cytology diagnostics process at Symbiant’s three pathology laboratories. *Lab A* Alkmaar Medical Centre, *Lab B* Westfriesgasthuis Hoorn, *Lab C* Zaandam Medical Centre

Figure 1 demonstrates the routine cytology diagnostics process at Symbiant’s three pathology laboratories (Alkmaar Medical Centre, Westfriesgasthuis Hoorn and Zaandam Medical Centre). All cytological specimens were routinely prescreened for abnormalities by one or two cytotechnicians. Subsequently, the prescreened specimens are examined by either a general pathologist (in the Alkmaar and Hoorn laboratories) or both general pathologists and expert cytopathologists in the Zaandam laboratory.

Starting from January 1, 2012, a cytopathology expert team in the Zaandam laboratory began reviewing defined categories of clinical cytology specimens from the Alkmaar and Hoorn pathology laboratories, resulting in a consensus diagnosis. The team consisted of two expert cytopathologists (DSG and MV). A second review was performed blinded to the initial diagnoses. The following types of specimens were routinely sent for intradepartmental second review: difficult or suspicious cases and cases with a discrepancy between the general pathologists’ diagnoses, with the clinical presentation or with immunohistochemical stains.

The cytopathologists were either consulted before case sign-out, when the initial pathologist was unable to offer a preliminary diagnosis, or asked for a second opinion after preliminary sign-out. For the purpose of this study, the cases where the expert cytopathologists were consulted pre-sign-out were excluded from analysis. In the remaining cases, the initial diagnoses and the expert diagnoses were recorded and compared.

### Assessment of concordance between initial and expert diagnoses

We thereby retrospectively assessed all clinical cytopathology cases of 2012 and 2013 that had been reviewed by the expert cytopathologists and determined concordance between initial and expert diagnoses. We applied the same definitions for minor and major discordances as described by Lueck et al. and Bomeisl et al. [32, 33]. Minor discordance was defined as a 1-step deviation on the scale of ‘non-diagnostic, benign, atypical, suspicious and malignant’ without an effect on treatment or prognosis. Major discordance was defined as either a deviation of ≥2 steps on this scale or a discordance with effect on patient management or prognosis.

### Validation of diagnoses by comparison with histological follow-up

We validated initial and expert diagnoses by comparison with same-site histological follow-up diagnoses. The process of follow-up identification is explained in the following section. The diagnosis closest to the follow-up diagnosis was deemed correct. Non-diagnostic specimens, which had insufficient diagnostic material or were non-representative, were not validated by histological follow-up.

### Identification of cytohistologically discordant cases

As follow-up identification is a very time-consuming activity, a cytotechnician at Symbiant (HdL) developed the follow-up tool Follow Up application SYMbiant (FUSYM), which provides histological follow-up for cytology specimens by automating several steps in the process. FUSYM has been developed in Microsoft Visual Studio 2010 professional and was written in Microsoft Visual Basic.

The Netherlands employs a unique system whereby all reports from the Dutch pathology laboratories are stored in a central database (PALGA) via a local server. All histological examinations subsequent to the cytological examination were routinely extracted from PALGA and loaded into FUSYM. The following was coded while loading data: tissue type, organ, sampling region, sampling method and side. Furthermore, diagnoses were classified at three levels, known as diagnostic group (unknown, non-diagnostic, benign, atypia or malignant), main diagnosis (benign was subdivided into no abnormalities, benign lesion and benign neoplasm, and malignant was subdivided into suspicious and malignant neoplasm) and specific diagnosis. Subsequently, the actual follow-up examination from the same site as the cytology specimen (i.e. same organ and sampling region) was determined manually for every cytology specimen.

Finally, cytology and histology follow-up diagnoses were compared at the level of diagnostic group to determine the cytohistologic concordance rate and the number of ‘false-negative’ and ‘false-positive’ cytology diagnoses. Suspicious as well as malignant cytology diagnoses with a malignant histological follow-up were deemed concordant. Non-diagnostic cytology or histology specimens or specimens with a diagnosis of atypia were excluded from analysis.

### Retrospective double reading of a sample of cytohistologically discordant cases initially not undergoing double reading

To assess the quality of cytodiagnostics of the cases from 2012 to 2013 that did not undergo routine double reading, concordance with histological follow-up was determined. Of the cytohistologically discordant cases, we randomly selected a sample of 100 specimens from the Alkmaar pathology laboratory to be retrospectively reviewed by the expert cytopathologists, blinded to the original diagnosis, and recorded whether the expert diagnosis was concordant or discordant with the initial diagnosis. A discordant diagnosis was subdivided into (1) a change from benign to malignant, or vice versa, resulting in expert cytohistologic concordance; (2) a change from benign or malignant into atypia; or (3) a change into non-diagnostic.

### Statistics

Statistical analysis was performed using SPSS 20. Initial diagnoses and expert diagnoses were compared using the unweighted Cohen’s kappa (*K*) coefficient. A *K* value of 0.00–0.20 indicates a slight agreement, 0.21–0.40 a fair agreement, 0.41–0.60 a moderate agreement, 0.61–0.80 a substantial agreement and 0.81–1 a perfect agreement. Furthermore, the percentages of concordance and discordance with their 95 % confidence intervals (CI) were calculated. *P* values <0.05 were considered statistically significant.

## Results

### Specimens routinely undergoing double reading

During the study period, 296 clinical cytology specimens underwent routine double reading by the expert cytopathologists. We excluded 78 cases where the initial diagnoses were not recorded, because pathologists consulted the expert team before case sign-out, leaving 218 specimens. From 12 patients, multiple cytology specimens were included as separate cases (11 patients with 2 specimens and 1 patient with 3 specimens). Table 1 summarizes the tissue types and sampling methods of the 218 specimens.

**Table 1:** Summary of tissue types and acquisition methods of 218 clinical cytology specimens undergoing double reading by expert cytopathologists

Tissue type	Number	Percentage
Thyroid FNA	52	23.9
Lymph node FNA	40	18.3
Pleural fluid	31	14.2
Salivary gland FNA	22	10.1
Bile duct brush	13	6.0
Urine	12	5.5
Bronchial FNA/brush/lavage	11	5.0
Breast FNA/nipple discharge	11	5.0
Ascitic fluid	8	3.7
Adrenal gland FNA	6	2.8
Liver FNA/brush	3	1.4
Pancreas FNA	2	0.9
Pericardial fluid	2	0.9
Cerebrospinal fluid	1	0.5
Peritoneal FNA	1	0.5
Esophageal FNA	1	0.5
Scrotal FNA	1	0.5
Retro-auricular FNA	1	0.5

*FNA* fine needle aspiration

Both diagnoses were concordant in 131 specimens (60.1 %; 95 % CI 0.535–0.666, kappa 0.489, *P* < 0.0001). Major discordance between the initial and the expert diagnoses was seen in 28 specimens (12.8 %; 95 % CI 0.084–0.173) and minor discordance in 59 specimens (27.1 %; 95 % CI 0.211–0.330). Table 2 summarizes the types of major and minor discordances. Of all discordant specimens, the initial diagnosis was underestimated 45 times (51.7 %) and overestimated 37 times (42.5 %). Twice, a benign diagnosis was changed into non-diagnostic, and 1 specimen was changed from non-diagnostic into malignant. Furthermore, for 1 specimen, an unspecific benign diagnosis (no malignancy) was specified into a Warthin tumour, and in another case, a diagnosis of metastatic squamous cell carcinoma was changed into metastatic adenocarcinoma.

**Table 2:** Types of major discordances (*n* = 28) and minor discordances (*n* = 59) for clinical cytology specimens undergoing double reading by expert cytopathologists

Type of major discordance	Number	Percentage	Type of minor discordance	Number	Percentage
Underestimated	14	50.0	Underestimated	31	52.6
Benign → suspicious	4	14.3	Benign → atypia	1	1.7
Benign → malignant	3	10.7	Atypia → suspicious	7	11.9
Atypia → malignant	7	25.0	Suspicious → malignant	23	39.0
Overestimated	13	46.4	Overestimated	24	40.7
Malignant → atypia	1	3.6	Malignant → suspicious	2	3.4
Malignant → benign	2	7.1	Suspicious → atypia	7	11.9
Suspicious → benign	10	35.7	Atypia → benign	15	25.4
Other	1	3.6	Other	4	6.8

Table 3 shows the frequencies of discordant second opinion diagnoses subdivided by the eight tissue types with ≥10 specimens reviewed by the expert cytopathologists. Pleural fluid, urine and bile duct brush specimens showed the highest overall discordance rates (58.1, 50.0 and 46.2 %, respectively). Major discordances were most commonly observed in pleural fluid, thyroid and urine specimens with 19.4, 19.2 and 16.7 %, respectively. Minor discordances were most commonly observed in bile duct brush, pleural fluid and bronchial specimens with 46.2, 38.7 and 36.4 %, respectively. Breast cytology specimens showed the lowest discordance rate, with one minor discordant expert diagnosis (9.1 %). The total major and minor discordance percentages of this subset of specimens (tissue types with ≥10 specimens) were similar to those of the whole study selection (all tissue types).

**Table 3:** Frequencies of discordances subdivided by tissue type

Tissue type	Number of specimens	Total discordant expert diagnoses	Major discordance	Minor discordance
Pleural fluid	31	18 (58.1 %)	6 (19.4 %)	12 (38.7 %)
Urine	12	6 (50.0 %)	2 (16.7 %)	4 (33.3 %)
Bile duct brush	13	6 (46.2 %)	–	6 (46.2 %)
Bronchial FNA/brush/lavage	11	5 (45.5 %)	1 (9.1 %)	4 (36.4 %)
Thyroid FNA	52	23 (44.2 %)	10 (19.2 %)	13 (25.0 %)
Lymph node FNA	40	11 (27.5 %)	6 (15.0 %)	5 (12.5 %)
Salivary gland FNA	22	6 (27.3 %)	–	6 (27.3 %)
Breast FNA/nipple discharge	11	1 (9.1 %)	–	1 (9.1 %)
Total	192	76 (39.6 %)	25 (13.0 %)	51 (26.6 %)

Tissue types with ≥10 cytology specimens reviewed by the expert cytopathologists were compared

*FNA* fine needle aspiration

### Validation by comparison with histological follow-up

Same-site histological follow-up was available for 25 of the 87 discordant specimens, but was non-diagnostic for 3 specimens. Hence, we validated the initial and expert diagnoses of 22 cytology specimens by comparison with histological follow-up. The expert diagnosis was supported by the histology diagnosis in 21/22 specimens (95.5 %; 95 % CI 0.860–1.049). The case that was not supported by histology revealed a malignant mesothelioma, which was not diagnosed in the pleural fluid cytology by both the initial pathologist as well as the expert cytopathologist.

### Retrospective second review of cytohistologically discordant cases

In order to get an impression of the quality of cytology diagnostics in those specimens that did not undergo double reading in routine diagnostics, we determined the rates of concordance and discordance with histological follow-up in these specimens. Same-site histological follow-up was available for 1613 cases, of which we excluded 338 cases, because either cytology, histology or both were non-diagnostic or had a diagnosis of atypia. Furthermore, we excluded 24 cases with a time period between cytological and histological examination longer than 6 months and 17 cases with only a few malignant cells on histology, leaving 1234 cases. Cytohistological concordance was found for 943 cytology specimens (76.4 %; 95 % CI 0.740–0.788), and 291 cytology specimens (23.6 %) had a discordant histological follow-up.

For the random sample of 100 cytohistologically discordant cases from the Alkmaar pathology laboratory, the expert diagnosis was consistent with the initial diagnosis in 57 % (95 % CI 0.471–0.669). The cytopathologists changed the diagnosis in 43 % of cases: in 17 cases, the diagnosis was changed from benign to malignant (10 cases) or vice versa (7 cases), resulting in cytohistological concordance; a benign or malignant diagnosis was changed into atypia in 8 cases, and a diagnosis was changed into non-diagnostic 18 times.

## Discussion

This study assessed the added value of our implemented intradepartmental double reading strategy of defined categories of clinical cytology specimens by a team of expert cytopathologists. We demonstrated a 60.1 % concordance rate, a 12.8 % major discordance rate and a 27.1 % minor discordance rate between initial and expert diagnoses. The highest major discordance rates were observed in pleural fluid, thyroid and urine specimens. Validation by comparison with same-site histological follow-up confirmed that expert diagnoses were correct in 95.5 % (95 % CI 0.860–1.049). These findings emphasize the importance of double reading of selected specimens by expert cytopathologists.

Previous studies on cytopathology double reading demonstrated somewhat lower major discordance rates (7.4 to 9.3 %) [32–34], probably due to differences in specimen selection. At our institution, defined categories of clinical cytology specimens were reviewed, whereas others described second review of all referred cytopathology material before definitive treatment. Furthermore, we specifically assessed the added value of double reading by expert cytopathologists. In these studies as well, high major disagreement rates of thyroid FNA specimens were observed (16.2 to 24.3 %), and in the study of Lueck et al. [32], major discrepancies in urine specimens were the third most common (16.2 %).

The high discordance rates in urine and pleural fluid specimens might be partly explained by the lack of standard terminology and the use of inadequate terms, especially for atypical lesions [35]. Implementation of the Paris System for Urinary Cytopathology, which is currently being developed, might improve urine cytology diagnostics [36]. This explanation does, however, not hold true for thyroid cytology specimens, because of well-defined terminology in the Bethesda System for Reporting Thyroid Cytopathology (BSRTC) [37]. We therefore suppose that most discrepancies were a result of inadequate interpretation instead of inadequate terms used. The majority of thyroid cytology discrepancies were caused by initial overestimation of benign and atypical specimens.

Initial underestimation occurred in slightly more discordant cytology specimens than overestimation did (51.7 and 42.5 %, respectively). This difference mainly represented minor discordant specimens, of which malignancies being underestimated as suspicious were most commonly observed, indicating reluctance among general pathologists to label cases as malignant. Among the major discordant specimens, the proportions of underestimated and overestimated diagnoses by general pathologists were evenly distributed.

A limitation of this study is the relatively small availability of same-site histological follow-up (in 25/87 discordant specimens) to validate expert diagnoses, which may lead to partial verification bias. Reasons for the absence of same-site histological follow-up were assessed. They included the presence of a benign cytology or benign follow-up cytology diagnosis (*n* = 18), the presence of histological follow-up obtained from another related site (*n* = 10) or radiological follow-up (*n* = 3). For 17 patients, histological follow-up would have been superfluous, because they already suffered from incurable metastatic malignancies. Furthermore, 12 patients were treated in an academic hospital or another local hospital, of which patient charts were unavailable to us, and 1 patient died very shortly after cytological examination. Finally, for 1 patient with an atypical thyroid cytology specimen, an intended hemithyroidectomy was probably cancelled for an, to us, unknown reason.

Our double reading strategy reveals major discordant diagnoses in a substantial number of cytology cases. Although standard double reading of all cytology specimens would be ideal in terms of patient safety, it would significantly increase workload and costs of pathology. Alternatively, all cytology could be signed out by expert cytopathologists, but this is in general pathology practice difficult to realize. In line with the present results, Raab et al. [38] demonstrated that focused review of diagnostically challenging areas of surgical pathology was more time- and cost-effective than 5 % random review and detected a significantly higher frequency of discrepancies. In order to get an impression of the quality of cytology diagnostics in those specimens that did not undergo double reading in routine diagnostics, we determined the concordance rate with histological follow-up, which appeared to be 76.4 %. Retrospective double reading of a random sample of 100 cytohistologically discordant specimens changed the diagnosis in 43 cases, with urine and lymph node specimens most commonly adapted. In these cases, the sign-out pathologist probably had been sufficiently confident of the diagnosis and therefore had not consulted the expert cytopathologists. This argues for investigating which further specimen types are problematic as well and would probably also benefit from initial double reading, in order to refine the double reading strategy.

Patient safety is of utmost importance, and, in our opinion, cytopathology is a subspecialization of pathology that requires specialized cytopathologists, because the discordance rates are unacceptably high. The Dutch thyroid carcinoma guideline [39] states that ‘thyroid FNAs should be assessed by a pathologist with interest and experience in thyroid cytology and histology, who can recommend management or treatment based on the cytology results. If an experienced pathologist is not available locally, the sample should be sent to a pathologist that does have expertise in this field.’ Also, the Dutch bladder carcinoma guideline [40] states that ‘reliability of urine cytology evaluation is dependent on the expertise of the (cyto)pathologist.’ The Board of Pathology of the European Union of Medical Specialists (UEMS) published requirements for recognition of postgraduate training in pathology and stated that cytopathology is an integral part of pathology and well-trained pathologists must be able to cover basic cytological diagnosis [41]. Pathologists can obtain post-graduate ‘advanced level of competence’ certificates in cytopathology [41]. We agree with Anshu et al. [42] who stated that ‘European and international guidelines for training and accreditation in cytopathology should be developed with some urgency’. Guidelines should include an annual minimum number of specimens that a (cyto)pathologist must view and recommendations for further education and examination.

## Conclusion

Our implemented double reading strategy of defined categories of cytology specimens showed major discordance in 12.8 % of specimens. The expert review was supported in 95.5 % of discordant cases where histological follow-up was available. This indicates that this double reading strategy is worthwhile and contributes to better cytodiagnostics and quality of patient care, especially for suspicious pleural fluid, thyroid and urine specimens. Although it is currently not reimbursed and formal cost-effectiveness studies are lacking, we believe that selected second review may prevent overtreatment of a subgroup of patients in a cost-effective way and, also in the light of the upcoming claim culture in Europe, should therefore be considered for regular reimbursement. Our results emphasize that cytopathology is a subspecialization of pathology and requires specialized cytopathologists.
